# Recent Advances in the Discovery and Function of Antimicrobial Molecules in Platelets

**DOI:** 10.3390/ijms221910230

**Published:** 2021-09-23

**Authors:** Alba S. Aquino-Domínguez, María de los A. Romero-Tlalolini, Honorio Torres-Aguilar, Sergio R. Aguilar-Ruiz

**Affiliations:** 1 Facultad de Medicina y Cirugía, Universidad Autónoma Benito Juárez de Oaxaca, Oaxaca 68020, Mexico; albasoledadaquinod@outlook.es; 2 Catedra CONACyT, Facultad de Medicina y Cirugía, Universidad Autónoma Benito Juárez de Oaxaca, Oaxaca 68020, Mexico; romerotlalolini@gmail.com; 3 Facultad de Ciencias Químicas, Universidad Autónoma Benito Juárez de Oaxaca, Oaxaca 68020, Mexico; qbhonorio@hotmail.com

**Keywords:** platelets, platelet microbicidal proteins (PMP), kinocidins, CHDPs (cationic host defense peptides)

## Abstract

The conventional function described for platelets is maintaining vascular integrity. Nevertheless, increasing evidence reveals that platelets can additionally play a crucial role in responding against microorganisms. Activated platelets release molecules with antimicrobial activity. This ability was first demonstrated in rabbit serum after coagulation and later in rabbit platelets stimulated with thrombin. Currently, multiple discoveries have allowed the identification and characterization of PMPs (platelet microbicidal proteins) and opened the way to identify kinocidins and CHDPs (cationic host defense peptides) in human platelets. These molecules are endowed with microbicidal activity through different mechanisms that broaden the platelet participation in normal and pathologic conditions. Therefore, this review aims to integrate the currently described platelet molecules with antimicrobial properties by summarizing the pathways towards their identification, characterization, and functional evaluation that have promoted new avenues for studying platelets based on kinocidins and CHDPs secretion.

## 1. Introduction

Platelets are anucleate cells ranging from 2–5 µm in size and are the most abundant cells in the blood circulation after erythrocytes [1]. Once released from their precursors, the megakaryocytes present in the bone marrow, platelets enter the bloodstream and circulate for 7 to 10 days [2]. Platelets are complex cells containing three different granules: the α, dense or δ, and lysosomal granules [3]. The α-granules carry diverse proteins, cytokines, chemokines, and growth factors, while the δ granules contain small molecules such as adenosine diphosphate (ADP), serotonin, glutamate, histamine, and calcium necessary for hemostasis [4]. Finally, lysosomal granules contain glycohydrolases and enzymes that degrade glycoproteins, glycolipids, and glycosaminoglycans [5]. Despite being anucleated cells, platelets contain stable messenger RNA (mRNA) transcripts and the translation machinery for protein synthesis due to the inheritance of their megakaryocytic precursors [6].

The principal described platelet function is their evident participation in hemostasis. In this process, platelets detect vascular damage by recognizing subendothelial components such as the von Willebrand factor (vWF) and collagen fibers through glycoproteins present on their surface. This recognition results in platelet activation and a hemostatic plug formed by platelet aggregation and fibrin deposits, avoiding blood loss [7,8]. In addition, to preserve vascular integrity, platelets own a broad functional repertoire of molecules participating in the immune system [9]. Platelets act as sensors of innate immunity because they present surface and intracellular pattern recognition receptors (PRRs) to recognize pathogen-associated molecular patterns (PAMPs) and damage-associated molecular patterns (DAMPs) [10]. Platelets also express complement receptors (CRs) [11], receptors for the crystallizable fraction of immunoglobulins (FcRs) [12], and receptors to recognize proinflammatory cytokines [13,14,15,16] and chemokines [17].

Additionally, activated platelets express on their surface ligands as CD62P, CD40L, and integrins, and secrete inflammatory mediators as IL-1β or chemokines that allow them to interact and induce certain functions in the innate immune response. Such functions include the respiratory burst, extravasation, transcription, cytokine release, the induction of neutrophil extracellular traps (NETs), or dendritic cell maturation [18]. In the adaptive immune response, platelets promote B lymphocyte isotype switching [19] and possess the molecular machinery for antigen presentation to cytotoxic T lymphocytes (CTL), including the immunoproteasome, the β2-microglobulin, and Major Histocompatibility Complex (MHC) class I molecules [20,21,22].

Outstandingly, platelets can also produce molecules with microbicidal activity. These particles include kinocidins (antimicrobial chemokines) [23,24] and cationic host defense peptides (CHDPs) [25]. In addition to antimicrobial properties, kinocidins and CHDPs are endowed with multiple biological functions expanding the platelet involvement in different physiological and disease-associated processes [26,27]. Therefore, this review aims to provide an overview of the discovery and identification of platelet-produced antimicrobial molecules, emphasizing recent advances and suggesting new avenues for investigating these undervalued cells.

## 2. Current Classification of Antimicrobial Molecules in Platelets

**Figure 1:**
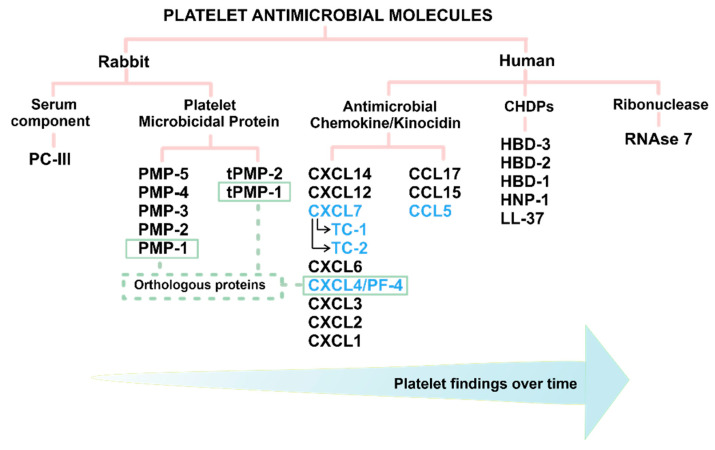
Classification of antimicrobial molecules in platelets. In rabbit and human platelets, antimicrobial molecules are chronologically grouped according to species, structure, or function (left to right). In rabbits, only a serum component (PC-III) and the platelet microbicidal proteins (PMP) not induced and induced by thrombin (tPMP) are found. PMP1 and tPMP-1 are orthologous with the human kinocidin CXCL4 or Platelet Factor 4 (PF-4). Some human kinocidins or kinocidin-derived fragments are primarily produced by platelets (highlighted in blue). In the group of cationic host defense peptides (CHDPs), both human neutrophil peptides 1 (HNP-1), human beta-defensins (HBD 1-3), and cathelicidin (LL-37) belong to this category. Currently, a single RNAse with antimicrobial activity (RNAse 7) is found in this classification.

The assigned terms to name antimicrobial molecules initially described in rabbit platelets and later in human platelets arose gradually over time, and their definition was coined depending on the species, structure, or functions performed (Figure 1). In 1981, the first serum component of peptide nature, with antimicrobial activity produced during the coagulation of rabbit platelets, was identified and called PC-III (third purification peak with antimicrobial activity obtained by column chromatography) [28]. In this same species, a group of not thrombin-induced platelet microbicidal proteins (PMP-1-5) was described within these cells, while the thrombin-induced secreted PMPs received the names tPMP-1, -2, demonstrating that PMPs and tPMPs are different from each other [29,30,31]. In humans, the first peptide purification with microbicidal activity from thrombin-stimulated platelets revealed chemokine CXCL7-derived fragments, called thrombocydins 1 and 2 (TC1 and TC2) [32]. The subsequent identification of more human microbicidal molecules revealed several CC and CXC chemokines families (CCL5, CXCL4, and CXCL7); hence, they were called kinocidins [33,34]. Although this name was initially used for platelet microbicidal chemokines, the term kinocidin now refers to microbicidal chemokines regardless of their cellular origin [35]. Between species, the human kinocidin CXCL4 or Platelet Factor 4 (PF-4) was described as an ortholog protein of PMP-1 and tPMP-1 in rabbits [34]. Finally, in the last ten years, other antimicrobial molecules termed CHDPs have been identified in human platelets; these particles include the human neutrophil peptides (HNPs), human β-defensins (HBD), and cathelcidin LL-37 [36]. The most recently described molecule with microbicidal function in human platelets is an RNAse-type enzyme (RNAse 7) [37].

## 3. Initial Findings of Antimicrobial Molecules in Platelets

Since the beginning of the 19th century, a thermostable substance with microbicidal activity against *Bacillus anthracis* was described in horse platelets. It was named plakin, whose bactericidal mechanism was described by inhibiting the cellular respiration [38]. D. M. Donaldson et al. [39] demonstrated that, by preventing rabbit platelet activation using anticoagulants in vivo or in vitro, treatment with X-irradiation, or the administration of antiplatelet antibodies, leads to a loss of the serum microbicidal capacity of these animals [39,40]. Interestingly, platelet-rich plasma (PRP) revealed more effectiveness than erythrocytes and isolated leukocytes to prevent *B. subtilis* growth. However, these microbicidal effects using human serum were slightly extended [41]. Subsequently, different assays using ethanol extractions identified two components responsible for the antimicrobial activity of rabbit serum; the sum of the two components was necessary to present this activity [40,42,43]. In the early 1980s, Carroll et al. [28,44,45] published a series of articles showing that the main thermostable factor responsible for the antibacterial activity against *B. subtilis*, *L. monocytogenes*, and *E. coli* present in rabbit serum is PC-III [44]. This component is a single peptide weighing 2000 daltons, composed of 17 amino acid residues, 24% basic and 34% nonpolar [28]. PC-III exerts a microbicidal activity against *B. subtilis* (the complete microorganism and its membrane vesicles). PC-III activity is mediated by a calcium-dependent mechanism capable of abolishing bacterial respiration in a lactate- and glucose-dependent manner, intervening in the respiratory complex that participates in the passage of the electrons from NADH to oxygen, by blocking the electron transport chain between cytochromes b and a, acting as an inhibitor of cytochrome c oxidase and cytochrome c reductase activity [45]. However, up to date, no further research has been conducted on this issue. 

## 4. Platelet Microbicidal Proteins of Rabbit: PMPs and tPMPs

In 1971, total platelet extracts, granules, and their secretion products obtained from activated rabbit platelets with collagen and ADP showed that they exert microbicidal activity against *B. subtilis* [46]. Approximately 20 years later, the thrombin-induced secretion products of rabbit platelets received the name of platelet microbicidal proteins (tPMP) [29,30,31]. The tPMP bactericidal action mainly occurs at 37 to 42 °C and pH 7.2 to 8.5. The presence of Na^+^, K^+^, Ca^2+^, and Mg^2+^ cations decreases tPMP microbicidal activity in a time- and concentration-dependent manner. In contrast, low-molecular-weight carbohydrates (glucose, sucrose, and melicytose) do not affect this function. The aforementioned indicates that tPMP has its highest microbicidal activity under conditions close to physiological [30]. tPMP can increase the effectiveness of the antibiotics oxacycline and vancomycin in killing *S. aureus* by reducing the minimum inhibitory concentration (MIC) and delaying the recovery time of microbial growth [31]. Similarly, tPMPs were found destroying different *C. albicans* strains and, to a lesser extent, *C. neoformans* through a mechanism involving cell surface structure damage [47]. In support of this idea, evaluating the response of tPMP-resistant strains of *C. albicans*, it became clear that they generate endocarditis with a higher density of fungal vegetation and dissemination to the spleen than rabbits treated with tPMP-susceptible strains [48]. Considering that pathogenic microorganism adherence to platelets can lead to hematogenous dissemination, the use of tPMP at sublethal doses conveniently demonstrated a decrease in platelet-*S. aureus* [49] and platelet-*C. albicans* interactions [50]. 

The characterization of rabbit platelet intracellular molecules through gel filtration and reverse-phase high-performance liquid chromatography (RP-HPLC) demonstrated the existence of five low-molecular-weight proteins with antimicrobial activity, which were assigned as PMP-1-5. On the other hand, two proteins were identified in the supernatant of thrombin-stimulated platelets using the same methodology and were called tPMP-1 and tPMP-2. PMPs and tPMPs are different from each other, and, until now, it has not been reported that PMPs 1-5 can be secreted or that tPMPs are present inside unstimulated rabbit platelets [51,52].

Using a protoplast model derived from tPMP-1 susceptible and resistant *S. aureus* strains, the importance of the bacterial growth phase was confirmed since the protoplasts of both resistant and susceptible bacterial strains showed the same tendency to membrane disruption during the logarithmic phase visualized by transmission electron microscopy (TEM) [53]. The bacteriolytic mechanism was demonstrated through artificial flat lipid bilayer membranes, where tPMP-1 addition caused an initial conductance increase with fluctuations from −30 mV to −90 mV and a voltage decrease to −30 mV; despite decreasing in voltage magnitude, the permeabilization was not abolished [53]. For tPMP-1 voltage-dependent action, the protein concentration is essential, as well as the polarity and extent of the membrane voltage, since permeabilization is even four times higher at a membrane voltage of −90 mV than at +90 mV, requiring a lower tPMP-1 amount to initiate permeabilization at −90 mV [54]. Furthermore, depending on the tPMP-1 quantity, the membrane damage pattern is different since low tPMP-1 concentrations correlate with a more significant effect on negative voltages in contrast to membrane damage at positive voltages related to high tPMP-1 concentrations [54]. Once the rabbit platelet proteins mechanism was decoded, it was compared with that exerted by HNP1. When analyzing the platelet microbicidal proteins (PMP and tPMP-1), substantial differences were found mainly due to environmental pH and critical membrane depolarization factors. In *S. aureus* strains with different membrane potentials, 6850 (Δψ −150 mV) and JB-1 (Δψ −100 mV), both PMP and tPMP-1 exert a membrane-potential-dependent mechanism, being more efficient against bacteria with a high membrane potential, 6850 (−150 mV), but at different pH, neutral for tPMP-1 and acidic for PMP-2. This is unlike HNP1, which showed a membrane-potential-independent mechanism eliminating both bacteria equally at a physiological pH [55].

On the other hand, antibiotics inhibiting cell wall synthesis (penicillin and vancomycin) increase the tPMP-1 ability to eliminate *S. aureus*. In contrast, bacteria pretreatment with drugs modifying intracellular processes by inhibiting protein synthesis (tetracycline, azithromycin, quinupristin), or DNA synthesis (novobicin), decrease its bactericidal capacity, suggesting that tPMP-1 also has intracellular targets [56].

Among the bacterial features providing specific resistance to tPMP-1-mediated elimination is a gene at the qacA locus of *S. aureus*. qacA encodes a proton motive force-dependent transporter, a pump exporting membrane-bound cations [57]. Another resistance mechanism employed by *S. aureus* strains is the phospholipid composition of the cytoplasmic membrane outer face. Phosphatidyl glycerol-lysine (LPG), a positively charged phospholipid, prevents tPMP-1 binding [58]. The tPMPs findings have been of great importance, the beginning of identifying human platelet microbicidal molecules.

## 5. Microbicidal Molecules in Human Platelets

### 5.1. Kinocidins 

The first group demonstrating a bactericidal substance released by thrombin-stimulated human platelets named it “thrombodefensins” in a doctoral thesis in 1988 [59]. Subsequently, this term was changed to “thrombocidins”. These molecules were identified as low-molecular-weight cationic peptides capable of exerting bactericidal activity against *S. viridans* [60]. The first human thrombocidins identification was made using cation exchange chromatography and continuous acid-urea polyacrylamide gel electrophoresis from the α-granules of platelets. This study found two peptides: thrombocidin-1 (TC-1) and thrombocidin-2 (TC-2). TC-1 and TC-2 characterization revealed that these peptides are derived from platelet basic protein (PBP, CXCL7) and are truncated variants in two amino acids (Ala-Asp) in the amino-terminal region of CXC chemokines: TC-1, from neutrophil-activating peptide 2 (NAP-2), and TC-2, from connective tissue activating peptide 3 (CTAP-3). Both components (TC-1 and TC-2) exerted potent bactericidal activity against *B. subtilis*, to a lesser extent against *E. coli* and *S. aureus*, and fungicidal activity against *C. neoformans* [32]. 

Subsequently, Tang et al. [33] purified seven platelet-derived antimicrobial peptides from supernatant resulting from thrombin stimulation or total platelet extract. In addition, using techniques such as RP-HPLC, mass spectrometry, amino acid analysis, and sequence determination revealed that the nature of the analyzed peptides corresponded to chemoattractant peptides (CXC and CC chemokines), previously characterized. Some identified chemokines were: platelet factor-4 (PF-4; CXCL4), Regulated on Activation Normal T cell Expressed and Secreted (RANTES or CCL5), CTAP-3, PBP, thymosin beta 4 (Tβ-4), fibrinopeptide (FP)-A, and FP-B. hPF4 and CTAP-3 are the most abundant peptides found in the secretion product of stimulated platelets. Additionally, the antimicrobial activity exerted by these isolated components was more potent against bacteria (*E. coli* and *S. aureus*) than against fungi (*C. albicans* and *C. neoformans*), mainly in acidic conditions (pH 5.5) [33]. 

**Figure 2:**
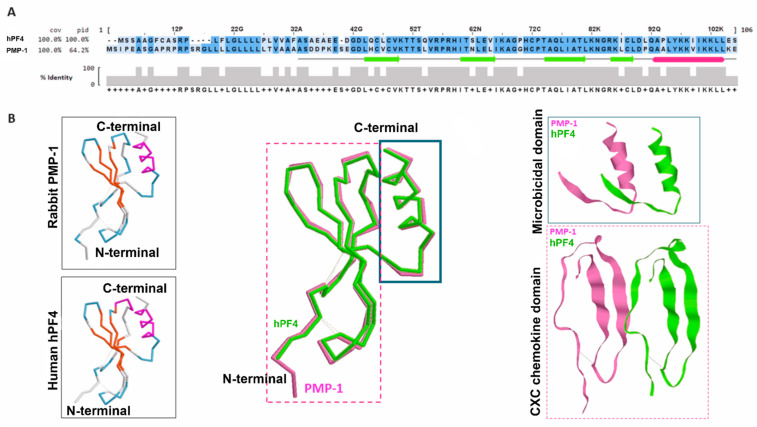
Comparative analysis of PMP-1 and human hPF4. (**A**) Comparative alignment of the primary structure of PMP-1 (rabbit) and hPF4 (human) generated with Clustal Omega and visualized with Jalview and Mview. hPF4 sequence was employed as a template. The total coverage percentage and identity are depicted on the left side. The predicted structure for PMP-1 with JPred of Jalview is indicated in the middle. The green arrows and red segment indicate the β-sheets and α-helices, respectively. The identity per base is represented in the gray scheme by the color intensity. (**B**) Left side, predicted structure for each protein obtained with SWISS-MODEL and visualized in RasMol in colors according to their structures. Both overlapping structures are visible in the center; the microbicidal domain is indicated in the solid blue line box and the chemokine domain in the dotted red line box. A dotted line indicates disulfide bonds. The human hPF4 is colored in green and PMP-1 of rabbit in pink. On the right side, a zoom of each domain. The chemokine domain was rotated to the right for more precise visualization of this structure.

The comparative analysis between rabbit and human microbicidal peptides showed that PMP-1 (ser-PMP-1, because of the serine presence at its N-terminal), and tPMP-1 (asp-PMP-1) present significant homology with human PF4 (hPF4) in their amino acid sequence (76% in the mature peptide), as well as their conformation, being considered ortholog proteins [34] (Figure 2A). Furthermore, using the hPF4 crystallographic structure as a basis [61], the following domains were identified in hPF4 as well as in ser-PMP-1 and Asp-PMP-1: (1) an N-terminal anionic region (from amino acid residue Ser1 or Asp1 to Arg25/24), with a CXC motif characteristic of chemokines; (2) an intermediate domain (from residue His25/26 to Arg 52/51), which conforms a β-sheet, antiparallel motif; (3) a C-terminal cationic domain (from residue Lys53/52 to Glu73/72), containing an α-helix motif consistent with peptides exhibiting direct microbicidal activity, and (4) a three-dimensional structure stabilized by two disulfide bridges [34] (Figure 2B). From this moment on, the term thrombocidins evolved towards another denomination that encompassed the main characteristics of these molecules, their chemotactic and microbicidal activity, to later be called kinocidins (*kino*- chemokine; *cidins*- microbicide) [35]. 

The domains that constitute the hPF4 and PMP-1 peptides have different functionalities: half molecule (residues 1–37) corresponds to the CXC chemokine domain, with a neutral charge and low antimicrobial activity, while the other half (residues 38-74) has a positive charge, an α-helix structure, and inhibits the growth of *S. aureus*, *S. typimurium*, and *C. albicans* [62] (Figure 2B). Despite the great hPF4 abundance in human platelets, there are few studies related to its antimicrobial (Table 1), viral (Section 6.1), and antiparasitic (Section 6.2) activity, and because hPF4 and PMP-1 are ortholog proteins, they might share similar chemotactic or antimicrobial mechanisms [62]. In addition to thrombin, the direct *S. aureus*/platelets interaction leads to kinocidins secretion through an ADP-dependent mechanism, in such a way that ADP degradation, or the use of antagonists of its receptors (P2X1 and P2Y12), prevents *S. aureus* clearance [63].

**Figure 3:**
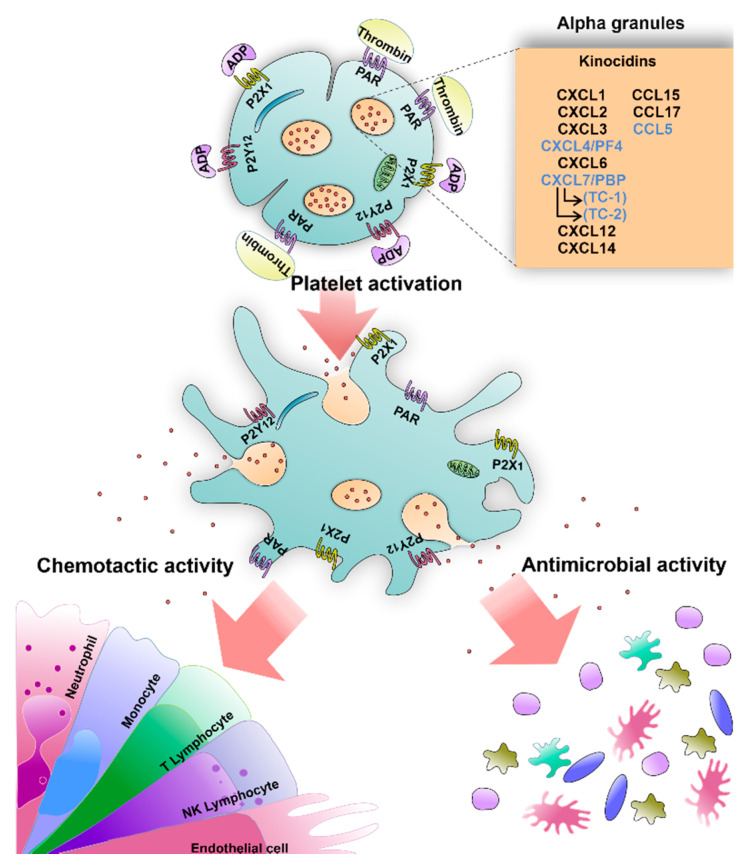
The activity of platelet antimicrobial chemokines/kinocidins. Nonactivated resting platelets store antimicrobial chemokines/kinocidins. Highlighted in blue in the box are those mainly produced by platelets (CXCL-4, CXCL-7, and CCL-5) and their derivatives (TC-1 and TC-2 from CXCL-7). After activation by agonists (ADP and thrombin), platelets release these molecules exerting dual functions, i.e., antimicrobial and chemoattractant for immune cells.

Finally, it is essential to mention some inconsistencies among different authors about the microbicidal activity of platelet kinocidins, maybe attributable to the experimental conditions or the microorganism type used in each investigation. For example, the studies performed by Cole AM et al. did not detect the antimicrobial capability of the chemokine RANTES against *E. coli* and *L. monocytogenes* at neutral pH [64]. The findings of Yang et al. ruled out the antimicrobial function of RANTES against *E. coli* and *S. aureus* at pH 7.4. Nevertheless, despite these variations, the authors detected variable antimicrobial activity against these pathogens using other chemokines identified in platelets: CXCL1, CXCL2, CXCL10, CXCL12, CXCL14, CCL17, and CCL20 [65]. Because there are more kinocidins in platelets, a complete list is included in Table 1 that summarizes their microbicidal activity and details their presence in human platelets. In conclusion, platelets can be activated by vascular damage signals (thrombin and ADP) or by direct microorganism recognition, leading to kinocidin releasing, which has microbicidal and chemoattractant functions for the immune system cells (Figure 3).

**Table 1:** Kinocidins in human platelets.

Chemokine	Platelet Findings	Target Microorganism
CXCL1	Presence of mRNA [66,67]	*E. coli*, *S. aureus*, *S. typhimurium* and *C. albicans* [35,65]
CXCL2	Proteomic analysis [68] and RNA sequencing [69]	*E. coli* and *S. aureus* [65]
CXCL3	Proteomic analysis [68] and RNA sequencing [69]	*E. coli* and *S. aureus* [65]
CXCL4	Isolation and characterization [70] and release by stimulation [71]	*E. coli*, *S. aureus*, *S. typhimurium* and *C. albicans* [35,62]
CXCL6	Proteomic analysis [68]	*N. gonorrhoeae*, *E. faecalis*, *P. aeruginosa*, *S. pyogenes*, *S. dysgalactiae subsp*, *S. aureus*, *E. coli*, and *B. subtilis* [72,73]
CXCL7	Purification of the secretion product and sequence analysis [33]	*E. coli*, *S. aureus* and *C. neoformans* [33]
CXCL7 (fragment TC-1)	Purification from granule-α and sequence analysis [32]	*E. coli*, *B. subtilis*, *C. neoformans* and *S. aureus* [32,74]
CXCL7 (fragment TC-2)	Purification from granule-α and sequence analysis [32]	*E. coli*, *S. aureus* and *B*. *subtilis* [32]
CXCL12	Protein expression and release by stimulation [75,76]	*E. coli* and *S. aureus* [65]
CXCL14	Surface expression and release by stimulation [77]	*E. coli*, *S. aureus*, *E.coli* and *C. albicans* [65,78]
CCL5	Expression of mRNA [66] and release by stimulation [79]	*E. coli*, *S. aureus* and *S. typhimurium*, [33,35]
CCL15	Release by stimulation [80]	*E. coli*, *S. aureus* [33]
CCL17	Release by stimulation (in vitro) and during coagulation [80,81]	*E. coli*, *S. aureus* [33]

### 5.2. Cationic Host Defense Peptides (CHDPs) in Platelets 

Cationic host defense peptides (CHDPs), also known as antimicrobial peptides (AMPs), are small, amphipathic peptides with less than 50 amino acids and a net positive charge of +2 to +9 at physiological pH [82]. Cationic CHDPs primarily target anionic bacterial membranes rich in phosphatidylglycerols; membrane-bound peptides can have several mechanisms, including causing lipid clustering and membrane permeabilization, or even membrane damage from carpet/toroidal models [83]. Membrane permeabilization of target cells is an important event necessary for translocating certain CHDPs into their cytoplasm. Furthermore, CHDPs target essential cellular processes, including DNA/RNA and protein synthesis, protein folding, enzymatic activity, and cell wall synthesis [84].

The two main CHDPs in vertebrates are defensins and cathelicidins, which are synthesized as immature peptides and, upon enzymatic cleavage, yield active peptides. Defensins have a common β-laminin core, stabilized with three disulfide bridges between six conserved cysteine residues, and are categorized into α-, β- and θ -defensins, based on their cysteine residue bonds [36]. α-defensins are found only in some mammals, primarily rodents and humans (HD, human defensins), where they have high expression in neutrophils, hence α-defensins are also known as human neutrophil peptides (HNPs), corresponding to HNPs 1-4. Additionally, in the human small intestine, Paneth cells express the α-defensins HD-5 and -6 [85,86,87]. β-defensins are ubiquitous and present in all vertebrates; humans have more than 30 genes coding for β-defensins, mainly expressed in epithelial cells [85]. Finally, cyclic θ-defensins have been identified in non-human primates [88]. 

**Figure 4:**
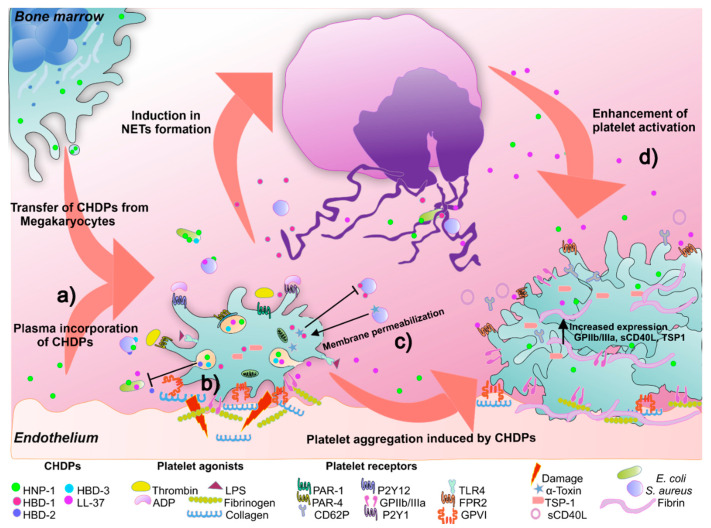
Platelet immune and hemostatic functions through cationic host defense peptides (CHDPs). (**a**) Platelet CHDPs arise as part of the arsenal inherited by their precursor cells or from extracellular uptake from plasma. (**b**) Some CHDPs are stored within granules and are released upon recognizing activation-inducing components exposed during vascular damage (collagen, thrombin, and ADP) or by the presence of microbial components (lipopolysaccharide). Taken together, platelet CHDPs are capable of eliminating a great diversity of pathogens. (**c**) Platelets contain HBD1 in their cytoplasm, and the presence of permeabilizing agents on the bacterial membrane (e.g., α-toxin) induces its release. HBD1 released by platelets kills bacteria directly or through NETs induction. (**d**) CHDPs of platelet originated or produced by other cells increase platelet activation (increased CD62P, GPIIb/IIIa surface expression, or TSP-1 and CD40L secretion), particularly for LL-37, through recognizing FPR2 receptor. Human neutrophil peptide-1 (HNP-1), human beta defensin 1-3 (HBD1-3), adenosine diphosphate (ADP), protease-activated receptors-1, -4 (PAR-1, -4), thrombospondin (TSP-1).

The scientific knowledge of CHDPs in human platelets has significantly increased in the last ten years because they have become noticeable in these cells. Tohidnezhad et al. showed that human platelets contain β-defensins (HBD)-2 [89] and HBD-3 [90] and can secrete them after thrombin stimulation. This supernatant is microbicidal for multiple bacterial strains. However, only HBD-3 contribution against *E. coli* and *P. mirabillis* was demonstrated [89]. On the other hand, HBD-1 was also found in platelets at mRNA and peptide levels outside α-granules; stimuli promoting platelet degranulation do not favor HBD1 release. Yet, HBD1 release can be induced by α-toxin from *S. aureus*, a platelet membrane permeabilizing molecule [91]. Impressively, platelet HBD-1 suppresses *S. aureus* growth and triggers neutrophil extracellular traps (NETs) generation [91]. Our research group described for the first time that platelets and megakaryocytes express both mRNA and HNP-1 peptide; the latter is found within platelet α-granules, and stimuli promoting platelet degranulation such as thrombin, recognized by PAR (protease-activated receptors)-1, -4; lipopolysaccharide (LPS), recognized by TLR4; and ADP, recognized by P2Y1 and P2Y12 receptors induce HNP-1 secretion [92]. Likewise, an in vitro differentiation system revealed that megakaryocytes inherit both mRNA and HNP-1 peptides to nascent platelets, which can also absorb this peptide from plasma. Importantly, platelet HNP-1 showed a relevant microbicidal function by inhibiting *E. coli* growth [92] (Figure 4).

Cathelicidins are also produced as immature peptides containing an amino-terminal signal peptide, a cathelin-like domain, and a mature peptide at the carboxyl-terminal. Once the peptide is secreted, the cathelin-like domain is cleaved by serine proteases [93]. The only human cathelicidin is LL-37, one of several cleavage products of human cationic antimicrobial protein 18 Kda (hCAP18), a product of the *CAMP* gene [94]. LL-37 has a broad inhibition spectrum, including bacteria, fungi, and viruses [95]. Interestingly, Salamah MF et al. identified LL-37 inside human platelets and showed that agonists such as collagen, selective glycoprotein VI (GPVI) agonist (CRP-XL), and thrombin receptor activating peptide 6 (TRAP6) could induce its release [96]. In addition, LL-37-treated platelets form more platelet-neutrophil aggregates, which in turn cause neutrophil activation, characterized by increased CD11b integrin expression and reactive oxygen species (ROS) [97] (Figure 4). Nevertheless, the relevance of platelet-derived LL-37 as a direct antimicrobial peptide needs further investigation. Given that megakaryocytes share the common myeloid progenitor (CMP) with neutrophils and monocytes, the presence of LL37 and other CHDPS in peripheral blood platelets might derive from megakaryopoiesis [98,99]. 

An aspect of great interest is that CHDPs expressed in human platelets can induce platelet activation and aggregation. HNP-1 has different effects on these cells, including increased binding to fibrinogen, augmented surface expression of activated glycoprotein GP IIb/IIIa, thrombospondin 1 (TSP-1), CD62P, CD63, secretion of soluble CD40L, and induction of platelet aggregation by forming amyloid-like structures, which can bind microorganisms [97]. Several HNP-1-induced effects on platelets are also triggered by LL-37, as human platelets express the LL-37 receptor, FPR2 (N-formyl peptide receptor 2)/AXL [96,100] (Figure 4). Endogenous LL-37 is of great importance for normal platelet activation because treatment of these cells with WRW4, an FPR2/AXL antagonist, inhibits the activation of agonist (CRP-XL or ADP)-treated platelets, and bleeding times are longer in Fpr2/3-deficient mice (an orthologous receptor for FPR2/AXL) [96]. Concerning these findings, LL-37 is abundant in thrombi of acute myocardial infarction patients, as well as CRAMP (a homologous molecule to LL37 in mice) in a model of carotid artery injury [100]. In addition to endogenous platelet CHDPs significance, it is necessary to consider the supply of neutrophil-derived CHDPs and a bidirectional platelet–neutrophil activation axis from CHDPs. In this sense, the importance of the adoptive transfer of CRAMP-treated platelets favoring neutrophil extravasation to sites of traumatic inflammation has been demonstrated [100].

### 5.3. RNAse 7

The most recent report of platelet antimicrobial molecules revealed that these cells express RNase 7 in basal conditions or when infected with *M. tuberculosis*, showing its localization within platelets by electron microscopy. Likewise, the authors detected the presence of hPF4 and HBD-2. Despite this discovery, the results indicate that platelets do not show direct antimycobacterial activity but secrete higher amounts of the proinflammatory cytokine IL-1β [37]. RNase 7 is an RNase with a cationic domain, which endows microbicidal activity against a wide range of pathogens. RNase 7 is primarily produced by epithelial cells, and different stimuli such as IL-17A, IFN-γ, IL-1β, and epidermal growth factor (EGF) increase its release [101]. In this sense, it is relevant to consider that platelets are the primary EGF producers in blood [102,103]; hence, through releasing this factor, increased RNase 7production might be induced in epithelial cells, being an indirect platelet mechanism participating in the innate immune response.

## 6. Platelet Antibacterial Molecules Participation against Other Infectious Agents

### 6.1. Antiviral Activity 

During viral infections, platelets are not only bystander cells in the organism because they exhibit alterations in their number and behavior [104,105]. Research has highlighted the platelets’ role in favoring virus and host damage [106]. However, there is also evidence of platelet contribution to virus elimination through various mechanisms, ranging from virus phagocytosis, ROS production, attraction, and activation of other immune cells [106] and even by direct antiviral processes throughout kinocidins participation [107]. hPF4, RANTES, and SDF-1 can suppress human immunodeficiency virus (HIV) infection in T lymphocytes by different mechanisms. hPF4 interacts with the external domain of the envelope glycoprotein gp120, preventing the virus entry process [108,109]. RANTES interacts with CD4 by blocking HIV binding to this receptor [110]. Finally, SDF-1 decreases CXCR4 surface expression, a coreceptor employed by HIV [111,112]. RANTES also participates in herpes simplex virus (HSV) elimination by interacting with its envelope glycoprotein (gB) and producing pores in the virions [113].

In addition to kinocidins, platelet-released CHDPs might perform an antiviral role. However, it has not been explored yet. The above due to HNP 1-3 have shown a broad antiviral activity against various viruses such as HIV [114], hepatitis C virus (HCV) [115], and human papillomavirus (HPV) [116]. Similarly, HBD-2 and -3 inhibit HIV [117] and vaccinia virus (VV) infections [118]. 

In this same sense, LL-37 has also shown a broad antiviral activity, including effects on several influenza virus strains [119], HCV [120], and dengue virus serotype 2 (DENV-2) [121]. In the most recent and alarming pandemic originating from SARS-CoV-2 [122,123], there is an evident platelet activation reflected by thrombocytopenia [124], high CD62P surface expression, reduced aggregation time after agonist treatment, and increased spreading on fibrinogen and collagen surfaces [125,126]. In COVID-19, the high hPF4 blood levels indicate platelet activation and degranulation [126]. However, at this time, the role of kinocidins and platelet-released CHDPs in SARS-CoV-2 infection remains unknown.

### 6.2. Antiparasitic Activity

Human platelets have been described as killing the responsible parasite of malaria (*P. falciparum*) without affecting the host’s erythrocytes [127]. This mechanism is mediated by platelet-secreted hPF4 and its interaction with Duffy antigen in the infected erythrocytes with *P. falciparum*. [128]. In this sense, the research of hPF4 antiparasitic effects against other protists such as *T. cruzi* or *Leishmania* species would be relevant. On the other hand, LL-37 has been located in biopsies of patients with leishmaniasis, and the exposure of *L. major* and *L. aethiopica* to recombinant LL-37 induces DNA fragmentation and death in a dose-dependent manner [129]. Similarly, LL-37 and its peptide fragments (KR-12, KR-20, and KS-30) reduce *E. histolytica* trophozoites integrity [130]. Likewise, the CHDPs LL-37, HBD1, and HBD2 reduce *C. parvum* sporozoites infectivity, whose effect increases when these CHDPs are combined with a neutralizing antibody targeting the apical complex of *C. parvum*, essential for its infective capability [131]. These findings suggest that platelet-derived kinocidins and CHDPs could play crucial protective roles in the pathogenesis of parasitic diseases.

## 7. New Non-Microbicidal Pathways for Platelet Antimicrobial Molecules

This last section explores some additional functions where platelet antimicrobial molecules can contribute. Beyond the microbicidal response, platelet kinocidins and CHDPs may also participate in other pathological processes such as cancer (explored in this section), autoimmunity [132], wound repair [133], and fibrosis [134]. The above aims to propose new possible platelet functions of their microbicidal molecules.

### 7.1. Immunoregulation

The relevance of platelet kinocidins is widely described, primarily due to their chemoattractant activity towards different leukocyte populations (monocytes, neutrophils, T and B lymphocytes, among others) [135]. In addition, kinocidins can regulate immune function in other ways. hPF4 inhibits interferon-α production in DENV-infected monocytes [136]. hPF4 and RANTES induce pro- and anti-inflammatory cytokines production in CD4 + T lymphocytes and their differentiation to Th1 and Th17 effector cells [137]. These facts highlight the relevant microbicidal role of platelet chemokines in immunoregulation and the need to search for new action mechanisms on immune cells.

There is now a better understanding of CHDPs ability for direct leukocytes recruitment. For example, HNP1 is a potent chemoattractant for T lymphocytes [138] and immature dendritic cells [139], whereas LL-37 is a chemoattractant for monocytes, neutrophils, eosinophils, and T lymphocytes, since these cell lineages express formyl peptide receptor-like 1 (FPRL1) [140,141]. Indirectly, LL-37 may favor leukocyte recruitment by increasing IL-1β and MCP-1 cytokines expression in whole blood and human epithelial cells [142], while HBD-2 and -3 stimulate keratinocytes to increase the expression and production of inflammatory cytokines IL-6, IP-10, MCP-1, MIP-3α, and RANTES [143]. Regarding the adaptive immune response polarization, some evidence shows that LL-37 and HBD-2, -3 can induce dendritic cells maturation and the subsequent Th1 lymphocytes generation [144,145,146]; similarly, HBD-2 and -3 favor plasmacytoid dendritic cells activation and the consequent IFN-α production [147]. Moreover, platelets express functional Toll-like receptors (TLRs)-1 to -10 [10], such that TLR4 stimulation with LPS in platelets and megakaryoblasts leads to HNP-1 secretion [92], whereas platelets stimulation with polyinosinic: polycytidylic acid (poly IC), the TLR3 ligand, leads to increased hPF4 secretion [148]. Parallelly, CHDPs can bind TLRs and modulate their activation, and LL-37 and HBD3 modulate the TLR4 signaling pathway, inhibiting inflammation in vitro and in vivo [149,150,151,152,153].

In sepsis patients, CHDPs levels are elevated in the bloodstream. This effect is associated with poor prognosis and, given that neutrophils are also present high numbers in this condition, CHDPs levels are deemed mainly derived from neutrophils secretion [154,155,156,157,158]. On the other hand, platelet activation in sepsis patients is evident by increased CD62P, TLR4, and protease-activated receptor 1 (PAR1) surface expression [159], as well as an increased hPF4 amount and platelet-derived microparticles [160]. In this scenario, platelets and megakaryocytes might contribute to these patients’ high CHDPs concentrations and the subsequent multifunctional effects mentioned above.

### 7.2. Anticancer Activity

CHDPs can also employ the membrane disruption mechanism described to kill bacteria to destroy cancer cells [161]. The above is due to detecting intracellular targets and altered qualities in the cancer cell membrane that distinguish them from normal cells [162,163,164]. HNPs disrupt the nucleus and membrane integrity [165] and counteract the proliferation of A549 cells (lung carcinoma) by inhibiting mitosis and angiogenesis. In addition, HNPs induce apoptosis and leucocyte infiltration to arrest tumor growth in a murine model [166], while HBD-1 and -3 act mainly by membrane permeabilization [167], leading to caspase-dependent apoptosis in tumor cells without damaging normal cells [168].

**Figure 5:**
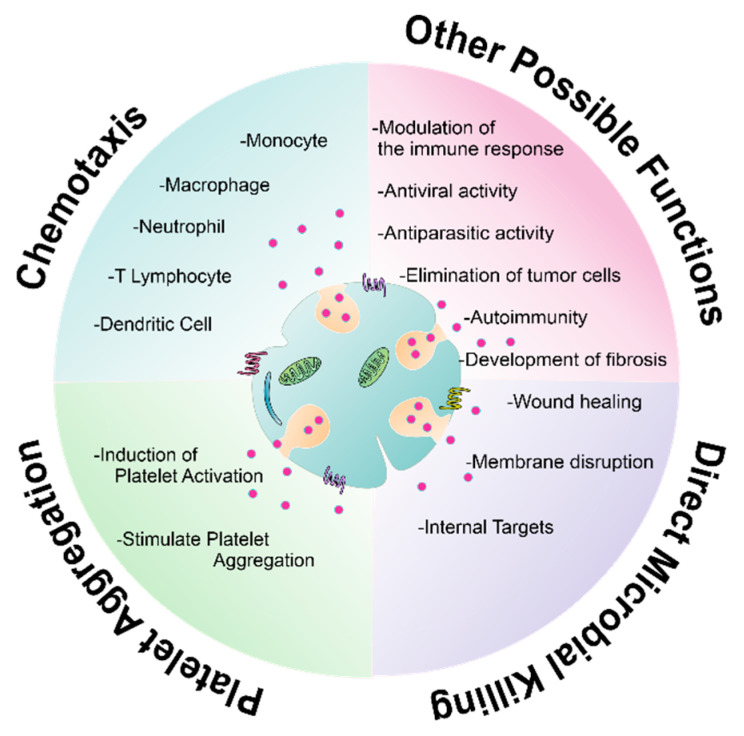
Functions described and probably exerted by antimicrobial molecules present in human platelets. Platelet-derived kinocidins and CHDPs participate in various scenarios, such as the direct microorganism killing, platelet activation and aggregation, and immune cells chemotaxis. Nevertheless, new contexts for and participation of platelet-derived antimicrobial molecules should be extensively explored, e.g., immunomodulation, antiviral and anticancer activity, among a plethora of potential functions.

LL-37 exhibits antitumor activity by inducing apoptosis and mitochondria membrane depolarization in oral squamous cell carcinoma cells (OSCC) without causing death in the immortalized human keratinocytes HaCaT cell line [169]. In addition, LL-37 can induce apoptosis of colon cancer cells as well as T lymphocyte tumor cells (Jurkat cells) in a calpain-dependent manner and nuclear translocation of the pro-apoptotic factors AIF (apoptosis-inducing factor) and EndoG (endonuclease G) through overexpression of p53-dependent members of the Bcl-2 family (Bax and Bak), triggering DNA fragmentation, chromatin condensation, loss of mitochondrial membrane potential and phosphatidylserine externalization (apoptosis signals) [170,171]. Finally, Figure 5 outlines the described functions of human platelet antimicrobial molecules and the new functions yet to be explored.

## 8. Conclusions

In the scenario described so far, platelet activation due to recognizing vascular damage signals, inflammatory mediators, or direct detection of infectious agents leads to releasing kinocidins and CHDPs. Such platelet activation products are per se endowed with direct microbicidal activity, and, additionally, they can act as chemoattractants for other immune cells and platelet aggregation inducers. However, there is still a long way to discover other molecules with antimicrobial activity in platelets and their impact on the immune response. Finally, the multifunctional characteristics of kinocidins and CHDPs secreted by platelets open new possibilities for the participation of these cells in different biological processes.

## Data Availability

Not applicable.
